# Supplementary material for: The interactome of intact mitochondria by cross-linking mass spectrometry provides evidence for coexisting respiratory supercomplexes
Source: Mol Cell Proteomics. 2017 Dec 8;17(2):216–32. doi: 10.1074/mcp.RA117.000470 (PMC5795388; doi:10.1074/mcp.RA117.000470)
Supplement: Supplemental Data [file supp_RA117.000470_133922_0_supp_23978_fzffwf.zip › spectra_annotation/mito_spectra_annotation/325-1-11-1-4-1.pdf]

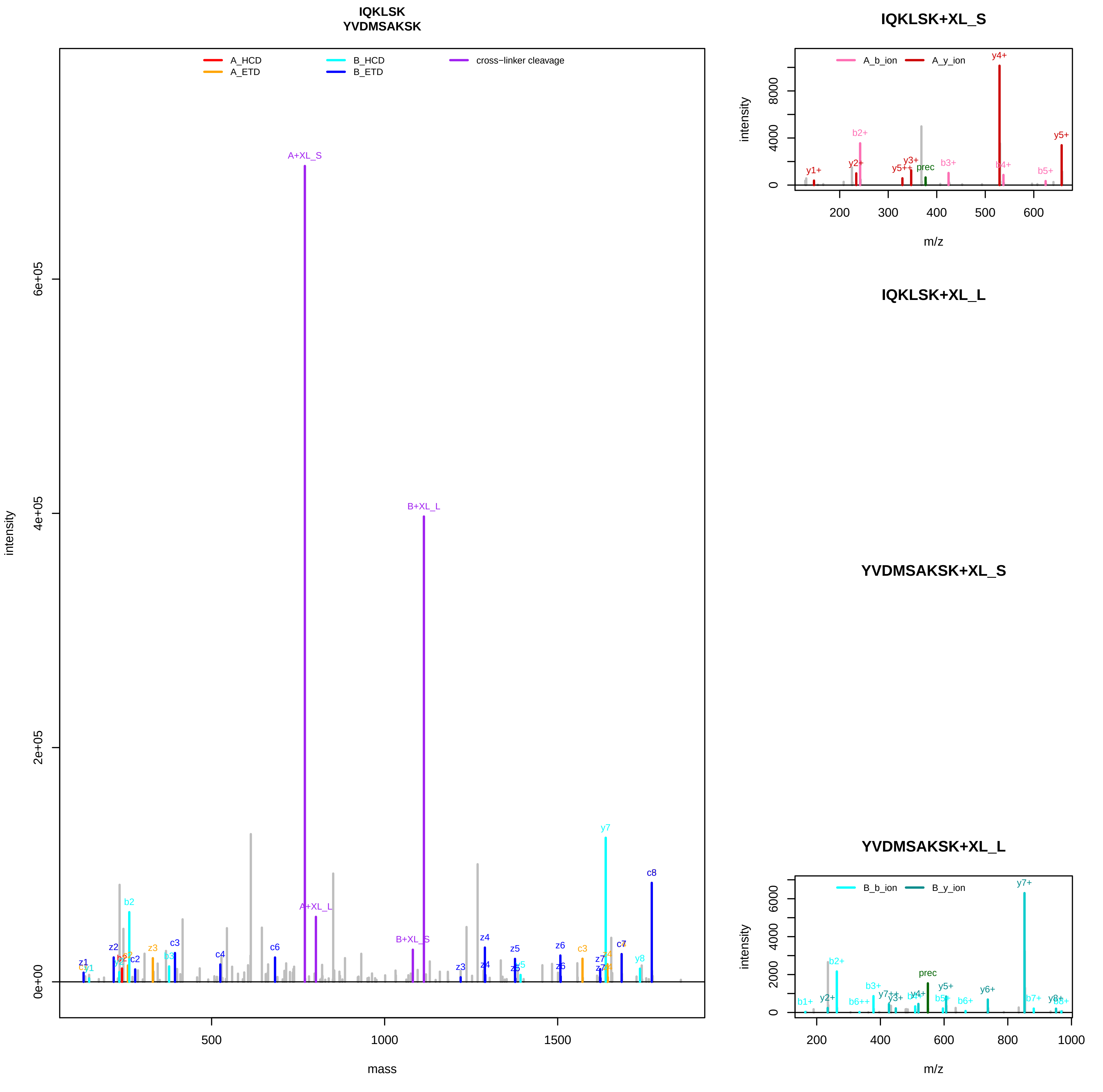
IQKLSK
YVDMSAKSK
A_HCD
A_ETD
B_HCD
B_ETD
cross−linker cleavage
A+XL_S
B+XL_L
A+XL_L
B+XL_S
intensity
mass
0e+00
2e+05
4e+05
6e+05
500
1000
1500
z1
z2
b2
c2
c3
c4
c6
z3
z4
z5
z6
z7
c7
c8
y7
y8
IQKLSK+XL_S
A_b_ion
A_y_ion
y1+
y2+
b2+
y5++
y3+
prec
b3+
y4+
b4+
b5+
y5+
m/z
0
4000
8000
200
300
400
500
600
IQKLSK+XL_L
YVDMSAKSK+XL_S
YVDMSAKSK+XL_L
B_b_ion
B_y_ion
b1+
y2+
b6++
b3+
y7++
y3+
b4+
y4+
prec
b5+
y5+
b6+
y6+
y7+
b7+
y8+
b8+
0
2000
4000
6000
200
400
600
800
1000
